# Perioperative Identification of an Accessory Fissure of the Right Lung

**DOI:** 10.1155/2015/954769

**Published:** 2015-06-22

**Authors:** Yannick Taverne, Gert-Jan Kleinrensink, Peter de Rooij

**Affiliations:** ^1^Department of Cardiothoracic Surgery, Erasmus Medical Center, 's-Gravendijkwal 230, 3015 CE Rotterdam, Netherlands; ^2^Department of Anatomy (ERCATHAN), Erasmus Medical Center, 's-Gravendijkwal 230, 3015 CE Rotterdam, Netherlands; ^3^Department of General Surgery, Maasstad Hospital, Olympiaweg 350, 3078 RT Rotterdam, Netherlands

## Abstract

Anatomical variations of lungs are common in clinical practice; however, they are sometimes overlooked in routine imaging. Surgical anatomy of the lung is complex and many variations are known to occur. A defective pulmonary development gives rise to variations in lobes and fissures. Morphological presentation is of clinical importance and profound knowledge of the organogenesis and functional anatomy is imperative for the interpretation and evaluation of lung pathophysiology and subsequent surgical intervention. However, appreciating them on radiographs and CT scans is difficult and they are therefore often either not identified or completely misinterpreted. As presented in this case report, an accessory fissure separating the superior segment of the right lower lobe from its native lobe was seen perioperatively and could only retrospectively be defined on X-rays and CT scan. It is imperative to keep in mind that accessory fissures can be missed on imaging studies and thus can make the surgical procedure more challenging.

## 1. Introduction

Anatomical variations of lungs are common in clinical practice and have been reported up to 40% in anatomical specimens [1, 2]. However, appreciating them on radiographs and CT scans is difficult and they are therefore either not identified or completely misinterpreted [1, 3]. Defective pulmonary development gives rise to variations in lobes and fissures which can only be comprehended from knowledge of embryology and developmental anatomy.

## 2. Case Report

We present a 33-year-old male admitted with progressive dyspnea and a nonproductive cough. Medical history includes an earlier admission with an atypical pneumonia (CURB 0: CURB-score is a clinical prediction rule that has been validated for predicting mortality in community-acquired pneumonia and infection of any site. In this case, CURB 0 represents a 30-day risk of death of 0.6%).

**Figure 1:**
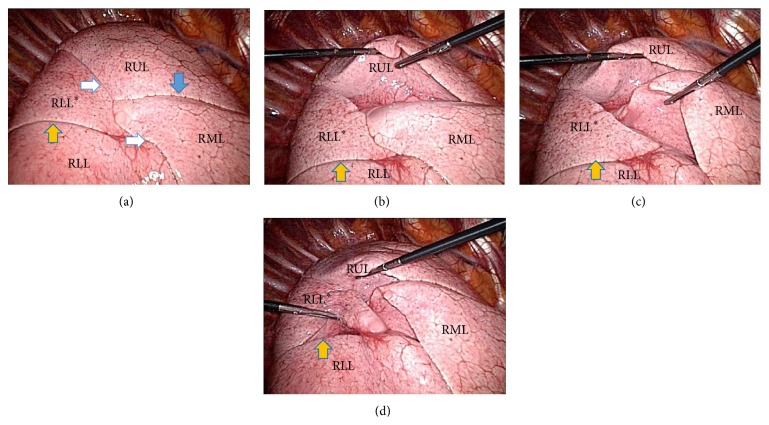
Perioperative pictures during VATS. All photographs were taken from the camera port distally with the patient lying on his left side and the arm in 90 degrees so the respective sides of these photographs concur with apical, ventral, distal, and dorsal positions, respectively. On the ventral side, the right internal mammary artery and vein running near the sternum can be seen. Pictures (a) to (d) display the different fissures as shown with long shaft instruments. RUL: right upper lobe; RML: right middle lobe; RLL: basal segments of the right lower lobe; RLL^*∗*^: apical segment of the right lower lobe. Solid white arrows: horizontal fissure; blue arrow: oblique fissure; orange solid arrow: superior accessory fissure of the right lower lobe.

**Figure 2:**
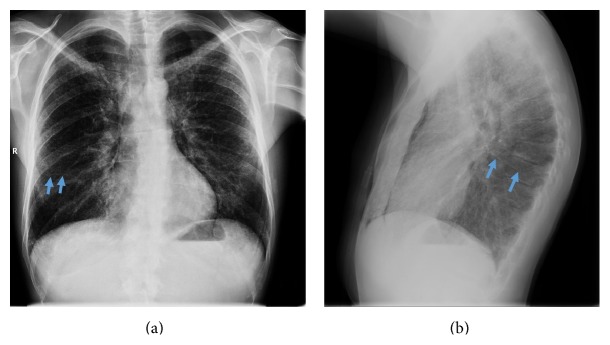
Plain X-rays. (a) anteroposterior X-ray; (b) lateral X-ray. Blue arrows show the superior accessory fissures (R = right side).

**Figure 3:**
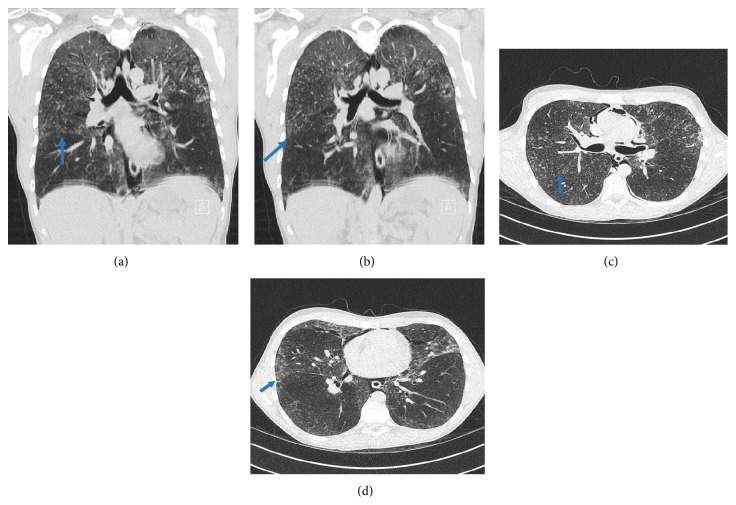
CT scan. (a)-(b) Coronal sections showing the bifurcation of the bronchus to the apical segment and the basal segments. There is no evidence of accessory branching, so no morphological extra lobe; hence there is only an accessory fissure (arrows). CT scans (a) and (b) have respiratory artifacts as seen at the level of the diaphragm. (c)-(d) Transversal sections showing the superior accessory fissure of the lower lobe of the right lung.

CT scan showed a pneumomediastinum with an interstitial lung disease without significant lymphadenopathy. Bronchoalveolar lavage and serology were negative. Therefore, video-assisted thoracoscopic (VATS) approach was used to obtain pulmonary biopsies. During VATS, we discovered an interesting anatomical variation of the lower lobe of the right lung; that is, the superior segment of the right lower lobe appeared separated from its native lobe through an extra fissure (Figures 1(a)–1(d)), thus giving this lung the image of a four-lobed organ. This finding was not visible on the plain X-ray (Figures 2(a)-2(b)) or detected during routine examination of the preoperative CT scan (Figures 3(a)–3(d)). Only after reexamination of the CT scan, the accessory fissure was detected.

## 3. Discussion

Fissures are defined as spaces separating individual bronchopulmonary buds or segments which get obliterated, except along two planes [4]. These planes will later be the horizontal or oblique fissure. When these spaces are not obliterated, accessory fissures of the lung are created. All variations in lobulation and fissures are the result of altered pulmonary development. The presence of a variant fissure can be due to partial or complete failure of obliteration of these fissures [5–7].

The most common accessory fissures of the right lung detected on CT scans are the inferior accessory fissure, demarcating the medial basal segment, and the superior fissure which defines the superior segment [1]. As in our case, a superior accessory fissure separates the superior segment of the lower lobe from the basal segment and is more common on the right side than on the left [8]. Superior accessory fissures have a reported incidence of 5–30% in autopsy studies as compared to the 3% incidence in high resolution CT scans [1, 5, 9]. In many cases, an accessory fissure fails to be detected on CT scan due to its incompleteness, thick CT scan sections, or orientation in relation to a particular anatomical plane [10].

From a functional and evolutionary point of view, a variant fissure separating the medial and lateral bronchopulmonary segments of the middle lobe and the basal segment from each basal segment, respectively, may be of advantage as it might limit the spread of infection [5]. It forms a sharp demarcated pneumonia which can be wrongly interpreted as atelectasis or consolidation [8]. Also, incomplete fissures are responsible for altering the spread of any lung disease [5].

## 4. Conclusion

It is imperative to keep in mind that an accessory fissure can be missed on imaging studies. Also, perioperative identification of the completeness of fissures and the presence of segmental localization is imperative before performing lobectomy. This is because individuals with an incomplete fissure are more prone to develop postoperative air leakage and thus possibly require further procedures such as a sleeve lobectomy.
